# Xuebijing alleviates acute hydrogen sulfide-induced pyroptosis in rat spleen via suppression of the caspase-1/NLRP3 pathway

**DOI:** 10.3389/fvets.2026.1914569

**Published:** 2026-07-29

**Authors:** Lulu Ding, Yuhao Han, Yan Chen, Hui Zou, Yonggang Ma, Xishuai Tong, Ruilong Song, Zongping Liu, Hongyan Zhao

**Affiliations:** 1 College of Veterinary Medicine, Yangzhou University, Yangzhou, China; 2 Jiangsu Co-Innovation Center for Prevention and Control of Important Animal Infectious Diseases and Zoonoses, Yangzhou University, Yangzhou, China; 3 Joint International Research Laboratory of Agriculture and Agri-Product Safety of the Ministry of Education of China, Institute of Agricultural Science and Technology Development, Yangzhou University, Yangzhou, China; 4 Research Facilities Sharing Center, Yangzhou University, Yangzhou, China

**Keywords:** hydrogen sulfide, NF-κB, NLRP3 inflammasome, pyroptosis, spleen, Xuebijing

## Abstract

Hydrogen sulfide (H_2_S) is a pervasive environmental toxicant with well-established immunotoxic effects. However, its action on lymphoid organs—particularly whether it triggers splenic pyroptosis—remains elusive. This study explored the effect of acute H_2_S exposure on rat spleen and the therapeutic potential of Xuebijing (XBJ), a traditional Chinese medicine for sepsis. Rats were exposed to 300 ppm H_2_S for 3 h, with or without 7-day XBJ pretreatment (4 mg/kg, i.p.). Splenic injury was evaluated by histopathology, ultrastructure, oxidative stress parameters, immunofluorescence, and immunoblotting. H_2_S exposure alone caused marked splenic damage, evidenced by increased organ coefficient, disrupted tissue architecture, mitochondrial swelling, and redox imbalance [depleted glutathione (GSH), superoxide dismutase (SOD), catalase (CAT); elevated malondialdehyde (MDA)]. Mechanistically, H_2_S activated the NF-κB/NLRP3/caspase-1 axis, upregulating NLRP3, ASC, cleaved caspase-1, and GSDMD-N, along with elevated interleukin-1β (IL-1β) and interleukin-18 (IL-18). XBJ pretreatment reversed these alterations, suppressing NF-κB phosphorylation, inflammasome assembly, and pyroptotic cell death. These findings demonstrate that H_2_S induces splenic pyroptosis via the NF-κB/NLRP3/caspase-1 pathway, and that XBJ confers protection by interrupting this cascade, offering a mechanism-based candidate for the treatment of acute H_2_S poisoning.

## 1 Introduction

Hydrogen sulfide (H_2_S) is a highly toxic gas with a characteristic odor of rotten eggs that is ubiquitously present in industrial settings, sewage systems, and sites of organic matter decomposition (1). Beyond its well-documented acute toxicity, recent large-scale environmental events have underscored the public health relevance of H_2_S exposure (2). As a significant environmental pollutant and occupational hazard, incidents of H_2_S poisoning are frequently reported, with severe cases rapidly leading to respiratory depression, loss of consciousness, and even death (3, 4).

The biological effects of H_2_S exhibit a well-established concentration-dependent duality (5). However, at supraphysiological concentrations encountered in environmental or occupational exposures, H_2_S overwhelms cellular detoxification pathways and exerts profound toxicity (6). The classical mechanism involves reversible inhibition of cytochrome c oxidase (mitochondrial complex IV), disrupting the electron transport chain, impairing cellular respiration, and ultimately leading to histotoxic hypoxia (7–9). Beyond this mitochondrial toxicity, H_2_S exposure triggers oxidative stress through multiple mechanisms, including depletion of intracellular glutathione, inactivation of antioxidant enzymes such as superoxide dismutase and catalase, and direct stimulation of NADPH oxidase-dependent reactive oxygen species (ROS) production (10). This oxidative imbalance, in turn, activates redox-sensitive transcription factors and promotes inflammatory gene expression. Accumulating evidence over the past decade has revealed that H_2_S can also trigger robust inflammatory responses and immunotoxicity, with studies documenting increased pro-inflammatory cytokine production, immune cell infiltration, and disruption of lymphoid organ architecture following exposure (11, 12). These findings warrant in-depth investigation into the mechanisms underlying H_2_S-induced damage to immune organs.

The spleen, as the largest secondary lymphoid organ, orchestrates both innate and adaptive immune responses and is particularly vulnerable to circulating toxins and inflammatory mediators (13, 14). Histopathological studies have demonstrated that H_2_S exposure induces marked structural disorganization in splenic tissue, characterized by disrupted lymphoid follicles, white pulp atrophy, lymphocyte depletion, and inflammatory cell infiltration (12). Nevertheless, the specific mode of programmed cell death underlying H_2_S-induced splenic injury remains to be fully elucidated. Pyroptosis is a lytic form of programmed cell death critically involved in host defense and inflammatory pathology (15). It is characterized by inflammasome assembly and caspase-1-dependent cleavage of gasdermin D (GSDMD), which releases its N-terminal fragment (GSDMD-N) (16, 17). The canonical NLR family pyrin domain containing 3 (NLRP3) inflammasome—a multi-protein complex comprising the sensor NLRP3, adaptor ASC, and effector pro-caspase-1—represents the core signaling axis regulating pyroptosis (18). Activation of NLRP3 requires two signals: a priming signal (signal 1) mediated by NF-κB-dependent upregulation of NLRP3 and pro-IL-1β, followed by an assembly/activation signal (signal 2) triggered by diverse stimuli including ROS, potassium efflux, and lysosomal damage (19–21). Recent studies have demonstrated that various environmental toxicants, including phthalates, nanoplastics, and heavy metals, can induce immune cell pyroptosis through NLRP3 inflammasome activation (20, 22–24). However, direct evidence regarding whether H_2_S mediates splenic cell pyroptosis via this pathway is currently lacking.

Xuebijing injection (XBJ), a compound traditional Chinese medicine preparation derived from five medicinal herbs—safflower (*Carthamus tinctorius*), red peony root (*Paeonia lactiflora*), Chuanxiong rhizome (*Ligusticum chuanxiong*), *Salvia miltiorrhiza*, and *Angelica sinensis*—originates from the classical Qing Dynasty formula “Yilin Gaicuo” (25, 26). Approved for market launch in 2004, XBJ remains the sole traditional Chinese medicine injection approved globally for the treatment of sepsis (27–29). High-quality clinical evidence, including the landmark EXIT-SEP trial and subsequent harmonized individual patient-level analyses, has demonstrated that XBJ significantly reduces 28-day all-cause mortality in patients with sepsis, particularly in those with septic shock or specific clinical phenotypes characterized by respiratory dysfunction (30–32). Mechanistic studies have revealed that XBJ exerts its therapeutic effects through a multi-component, multi-target paradigm, modulating key signaling pathways including TLR4/NF-κB, HMGB1/RAGE, and ROS-mediated NLRP3 inflammasome activation (33, 34). Zhang et al. (35) demonstrated that XBJ alleviates lipopolysaccharide-induced acute lung injury by inhibiting GSDME-mediated pyroptosis in alveolar epithelial cells, while Chen et al. (33) reported that XBJ suppresses M1 polarization of alveolar macrophages through regulation of ROS-mediated NLRP3 inflammasome signaling. Additionally, XBJ has been shown to reduce mortality in lung-injured mice by inhibiting HMGB1/RAGE axis-mediated pro-inflammatory cytokine secretion (36). The bioactive constituents responsible for these effects, including hydroxysafflor yellow A, paeoniflorin, and baicalein, have been progressively identified, with recent studies revealing that baicalein directly targets KEAP1 to activate the NRF2 antioxidant pathway (37–39).

Despite this growing body of evidence supporting XBJ's broad therapeutic potential in inflammatory conditions, its effects on H_2_S-induced immunotoxicity remain largely unexplored. This represents a significant knowledge gap, given that H_2_S poisoning remains a pressing occupational and environmental health concern with no specific antidote for its immunological consequences (40). Current management of H_2_S toxicity is largely supportive, focusing on respiratory support and removal from exposure, highlighting the urgent need for mechanism-based therapeutic interventions (41, 42). Importantly, the pathological processes triggered by H_2_S—oxidative stress, NF-κB activation, and NLRP3 inflammasome assembly—are precisely those known to be modulated by XBJ in other disease contexts (33, 35, 43). This mechanistic overlap provides a strong rationale for investigating whether XBJ can confer protection against H_2_S-induced splenic injury. We therefore hypothesized that XBJ may attenuate H_2_S-induced splenic damage through modulation of the caspase-1/NLRP3 pathway and subsequent inhibition of pyroptotic cell death. To test this hypothesis, the present study established a rat model of acute H_2_S poisoning and examined the effects of XBJ pretreatment on splenic histopathological damage, oxidative stress parameters, pyroptosis-related markers, and the expression of key molecules in the caspase-1/NLRP3 pathway. This study aims to elucidate the mechanism by which XBJ protects against H_2_S-induced immunotoxicity and provide experimental evidence supporting its potential clinical application in the prevention and treatment of H_2_S poisoning.

## 2 Materials and methods

### 2.1 Animals and experimental design

All animal procedures were approved by the Institutional Animal Care and Use Committee of Yangzhou University [Approval No. SYXK (Su) 2022-0044] and conducted in accordance with the guidelines of the National Institutes of Health Guide for the Care and Use of Laboratory Animals. Male Sprague-Dawley rats, aged 7 weeks with body weights ranging from 260 to 280 g, were obtained from the Comparative Medicine Center at Yangzhou University (Yangzhou, China). Throughout the experimental period, animals were maintained in an environment with controlled temperature (22 °C ± 2 °C) and relative humidity (50 ± 10%), under a 12-h light/dark cycle, and provided unrestricted access to standard rodent chow and water. Prior to initiating any experimental procedures, a 1-week acclimatization period was observed to allow adaptation to housing conditions.

Acute H_2_S poisoning was induced by controlled inhalation of H_2_S at a concentration of 300 ppm, following established protocols (44). Specifically, rats were placed inside a purpose-built 60-L glass chamber interfaced with a calibrated gas cylinder containing 1% (10,000 ppm) H_2_S balanced in nitrogen (Shangyuan Gas Company, China). Throughout the exposure period, H_2_S concentrations within the chamber were continuously monitored using a portable gas detector. Following the 3-h exposure, animals were returned to their home cages and observed for 12 h.

Rats were randomly divided into four groups (*n* = 4 per group): (1) Control: received daily intraperitoneal injections of normal saline for 7 days and were exposed to air; (2) XBJ: received daily intraperitoneal injections of XBJ (4 mg/kg body weight; Tianjin Hongri Pharmaceutical Co., Ltd., China) for 7 days and were exposed to air; (3) H_2_S: received daily intraperitoneal injections of normal saline for 7 days, followed by inhalation exposure to 300 ppm H_2_S for 3 h on day 7; (4) H_2_S+XBJ: received daily intraperitoneal injections of XBJ (4 mg/kg) for 7 days, followed by inhalation exposure to 300 ppm H_2_S for 3 h on day 7. The *n* = 4 per group was chosen based on two considerations. First, from a statistical perspective, it is generally accepted that *n* ≥ 3 is sufficient for basic parametric analyses when the intra-group variability is relatively low. Second, in accordance with the 3R principle (Replacement, Reduction, Refinement) and the guidelines of the Institutional Animal Care and Use Committee, we used the minimum number of animals required to obtain reliable and reproducible results while minimizing animal use and suffering (45, 46).

Upon completion of the 12-h post-exposure observation interval, all animals were deeply anesthetized via intraperitoneal administration of 2% sodium pentobarbital (30 mg/kg) and subsequently euthanized by exsanguination. Spleen tissues were harvested promptly for downstream analyses. Throughout the study, all efforts were made to minimize animal suffering and ensure welfare.

### 2.2 Measurement of body weight, spleen weight, and spleen coefficient

The body weight of each rat was recorded prior to euthanasia. After euthanasia, spleens were excised, rinsed with ice-cold phosphate-buffered saline (PBS), blotted dry, and weighed immediately. The spleen coefficient was calculated as: spleen weight (mg)/body weight (g) × 100% (47).

### 2.3 Determination of oxidative stress markers by ELISA

Splenic tissues were weighed and homogenized in ice-cold physiological saline solution (1:9, w/v) using a glass homogenizer. The homogenates were centrifuged at 12,000 × g for 15 min at 4 °C, and the resulting supernatants were collected for biochemical analyses. The levels of glutathione (GSH), malondialdehyde (MDA), catalase (CAT), and superoxide dismutase (SOD) were determined using commercially available assay kits following the manufacturer's protocols. All kits were obtained from the Jiancheng Bioengineering Institute (Nanjing, Jiangsu, China): GSH assay kit (Cat. No. A006-2-1), MDA assay kit (Cat. No. A003-1-2), CAT assay kit (Cat. No. A007-1-1), and SOD assay kit (Cat. No. A001-3-2). Protein concentrations in the supernatants were measured using the BCA protein assay kit (Cat. No. A045-4-2, Jiancheng Bioengineering Institute, Nanjing, China) to normalize the oxidative stress markers. All samples were analyzed in duplicate to ensure accuracy and reproducibility.

### 2.4 Hematoxylin and eosin (HE) staining

Spleen tissues were fixed in 4% paraformaldehyde for 24 h, dehydrated through a graded ethanol series, cleared in xylene, and embedded in paraffin. Sections of 4–5 μm thickness were cut, deparaffinized, rehydrated, and stained with hematoxylin and eosin (HE) according to standard protocols. Histopathological changes were observed under a light microscope at × 200 and × 400 magnifications. The degree of splenic injury was evaluated based on the disruption of splenic architecture, lymphocyte depletion, and inflammatory cell infiltration.

### 2.5 Transmission electron microscopy (TEM)

For ultrastructural observation, fresh spleen tissues were cut into small pieces (approximately 1 mm^3^) and fixed in 2.5% glutaraldehyde in 0.1 M phosphate buffer (pH 7.4) at 4 °C for 4 h. After washing with phosphate buffer, samples were post-fixed with 1% osmium tetroxide for 2 h, dehydrated through a graded acetone series, and embedded in Epon 812 resin. Ultrathin sections (70 nm) were cut using an ultramicrotome, stained with uranyl acetate and lead citrate, and examined under a transmission electron microscope at an accelerating voltage of 80 kV. Morphological features of pyroptosis, including plasma membrane rupture, chromatin condensation, and mitochondrial swelling, were assessed.

### 2.6 Immunofluorescence staining

Immunofluorescence staining was performed to detect the expression and localization of NLRP3 and caspase-1 in spleen tissues. Paraffin-embedded spleen sections (4 μm) were deparaffinized, rehydrated, and subjected to antigen retrieval by heating in citrate buffer (10 mM, pH 6.0) for 15 min. After blocking with 5% bovine serum albumin (BSA) in PBS for 1 h at room temperature, sections were incubated overnight at 4 °C with primary antibodies: rabbit anti-NLRP3 polyclonal antibody (1:1000, Cat. No. 19771-1-AP, Proteintech, USA) and mouse anti-caspase-1 monoclonal antibody (1:1000, Cat. No. 22915-1-AP, Proteintech). After washing with PBS, sections were incubated with Alexa Fluor 647-conjugated goat anti-rabbit IgG (1:500, Cat. No. A0468, Proteintech) for 1 h at room temperature in the dark. Nuclei were counterstained with 4′,6-diamidino-2-phenylindole (DAPI, Cat. No. D9542, Sigma-Aldrich, USA). Representative images were captured from at least five randomly selected fields per section using a confocal laser scanning microscope and processed with ImageJ software.

### 2.7 Western blot analysis

To assess the expression levels of proteins involved in the NF-κB and NLRP3/caspase-1 signaling pathways, western blotting was carried out. Splenic tissues were homogenized in RIPA lysis buffer supplemented with protease and phosphatase inhibitor cocktails, and the lysates were clarified by centrifugation. Protein content in the supernatants was quantified using a BCA assay. Equivalent amounts of protein (30–50 μg per lane) were resolved by 10%−12% Sodium dodecyl sulfate-polyacrylamide gel electrophoresis (SDS-PAGE) and subsequently electrotransferred onto Polyvinylidene fluoride (PVDF) membranes. After transfer, the membranes were saturated with 5% non-fat milk in TBST for 1 h at ambient temperature, followed by overnight incubation at 4 °C with primary antibodies directed against NF-κB p65, phospho-NF-κB p65 (Ser536), IκBα, phospho-IκBα (Ser32), IκBα, NLRP3, ASC, caspase-1, cleaved caspase-1 (p20), GSDMD, cleaved GSDMD (GSDMD-N), IL-1β, and IL-18. After thorough washing, the membranes were incubated with HRP-conjugated secondary antibodies (goat anti-rabbit or goat anti-mouse) for 1 h at room temperature. Immunoreactive bands were visualized using enhanced chemiluminescence reagents and recorded with a chemiluminescence imaging system. Densitometric quantification was performed with ImageJ software, USA. GAPDH served as the loading control; for phosphorylated proteins, values were normalized to the corresponding total protein signal. Table 1 lists the antibodies utilized in this study.

**Table 1:** Antibodies used in the present study.

Antibody	Catalog number	Company
NLRP3	19771-1-AP	Proteintech
GSDMD	39754	CST
Cleaved GSDMD	36425	CST
Caspase-1	2225	CST
Cleaved caspase-1	89332	CST
ASC	10500-1-AP	Proteintech
NF-κB	86652	CST
p-NF-κB	3033	CST
IκBα	4812	CST
p-IκBα	2859	CST
IL-18	10663-1-AP	Proteintech
IL-1β	16806-1-AP	Proteintech
GAPDH	60004-1-Ig	Proteintech
HRP-conjugated goat anti-rabbit IgG (H+L)	P8002	NCM Biotech
HRP-conjugated goat anti-mouse IgG (H+L)	P8001	NCM Biotech

### 2.8 Statistical analysis

All experimental data were expressed as mean ± standard deviation (SD) derived from a minimum of three independent biological replicates. Statistical evaluations were conducted using GraphPad Prism 9.0 software (USA). For comparisons involving multiple groups, one-way analysis of variance (ANOVA) was applied, followed by Tukey's multiple comparison test to determine specific inter-group differences. A probability (*p*) value of less than 0.05 was considered to indicate statistical significance.

## 3 Results

### 3.1 XBJ ameliorates H_2_S-induced alterations in splenic coefficient and oxidative stress parameters

The experimental protocol is schematically depicted in Figure 1A. To evaluate the systemic effects of H_2_S exposure and XBJ intervention, body weight, spleen weight, and splenic coefficient were assessed across experimental groups. As shown in Figure 1B, no significant differences in body weight were observed among the experimental groups throughout the study period, indicating that H_2_S exposure and XBJ pretreatment did not substantially affect overall growth under the current experimental conditions. However, spleen wet weight was significantly increased in the H_2_S group compared with the Control group, an effect that was markedly reversed by XBJ administration (Figure 1C). Consequently, the splenic coefficient—calculated as spleen weight normalized to body weight—was substantially elevated following H_2_S exposure, whereas XBJ pretreatment significantly reduced this parameter (Figure 1D), suggesting that XBJ alleviates H_2_S-induced splenic edema or inflammatory enlargement independent of body weight changes.

**Figure 1:**
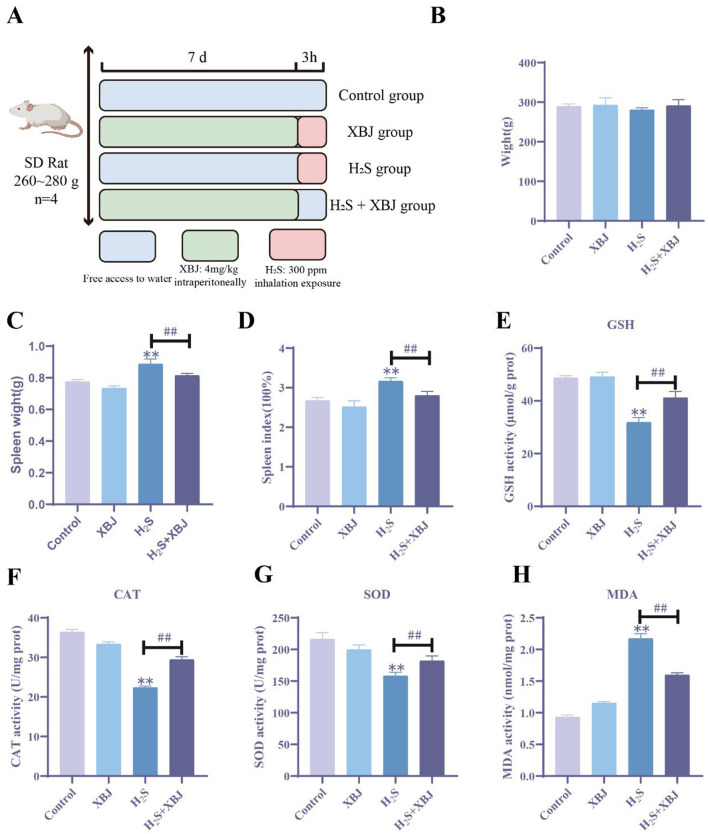
Effects of XBJ on splenic coefficient and oxidative stress parameters in H_2_S-exposed rats. **(A)** Schematic illustration of the experimental protocol. **(B)** Temporal changes in body weight across experimental groups (*n* = 4). **(C)** Spleen wet weight measured at 12 h post-exposure (*n* = 4). **(D)** Splenic coefficient calculated as spleen weight (mg) normalized to body weight **(g)** (*n* = 4). **(E)** GSH content in splenic homogenates. **(F)** CAT enzymatic activity. **(G)** SOD activity. **(H)** MDA levels as an index of lipid peroxidation. All oxidative stress markers were determined using commercial ELISA kits and normalized to total protein content. Data are expressed as mean ± SD (*n* = 4). **p < 0.01 vs. Control group; ^##^*p* < 0.01 vs. H_2_S group (one-way ANOVA followed by Tukey's *post hoc* test).

Oxidative stress parameters in splenic homogenates were measured to assess the redox imbalance provoked by H_2_S. As illustrated in Figures 1E–H, H_2_S exposure led to a significant depletion of antioxidant reserves, evidenced by reduced GSH content (Figure 1E), diminished CAT enzymatic activity (Figure 1F), and decreased SOD activity (Figure 1G) compared with the Control group. Concurrently, MDA levels—an indicator of lipid peroxidation—were markedly elevated in the H_2_S group (Figure 1H). These findings align with previous reports demonstrating that sulfide exposure induces considerable oxidative stress in various tissues through inhibition of mitochondrial respiration and generation of ROS (12, 33). Pretreatment with XBJ effectively restored GSH levels and antioxidant enzyme activities while suppressing MDA accumulation, as evidenced by significant differences between the H_2_S+XBJ group and the H_2_S group (Figures 1E–H), indicating that XBJ counteracts H_2_S-induced oxidative injury. This is consistent with the known antioxidant properties of XBJ, which have been attributed to its ability to downregulate oxidative stress via multiple signaling pathways (48).

### 3.2 XBJ attenuates H_2_S-induced histopathological damage in splenic tissue

Gross morphological examination revealed notable splenomegaly in the H_2_S group compared with controls, an effect that was less pronounced in the H_2_S+XBJ group (Figure 2A). Histological assessment via hematoxylin and eosin (HE) staining demonstrated that spleens from Control and XBJ-treated animals exhibited normal microarchitecture, with clearly demarcated white pulp and red pulp regions (Figure 2B). In contrast, H_2_S exposure induced severe structural disorganization, characterized by disrupted lymphoid follicles, white pulp atrophy, and extensive inflammatory cell infiltration. These histopathological alterations were markedly ameliorated in the H_2_S+XBJ group, with partial restoration of splenic architecture.

**Figure 2:**
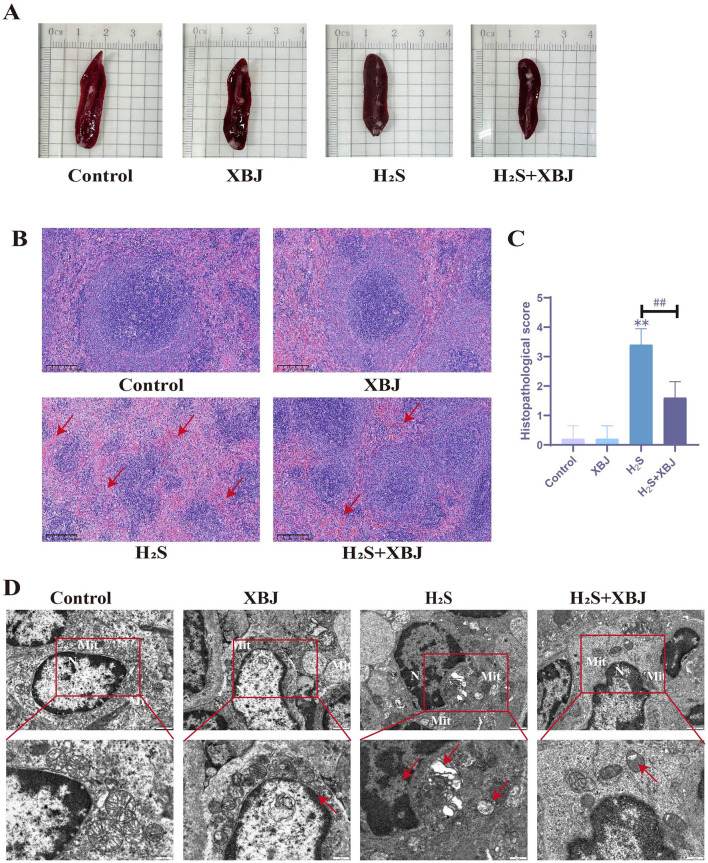
XBJ attenuates H_2_S-induced histopathological alterations in splenic tissue. **(A)** Representative gross morphology of spleens harvested from each experimental cohort. **(B)** HE staining depicting microarchitectural changes in splenic sections; red arrows indicate the characteristic pathological changes induced by H_2_S exposure. Scale bar = 200 μm. **(C)** Semiquantitative histopathological scoring of splenic injury based on a 0–4 scale (0, normal; 4, severe damage with extensive structural disorganization). **(D)** TEM images revealing ultrastructural features of pyroptosis, including mitochondrial swelling with vacuolization and disruption of cristae (red arrowheads). Scale bar = 1 μm. **p < 0.01 vs. Control group; ^##^*p* < 0.01 vs. H_2_S group.

Semiquantitative scoring of splenic injury confirmed these observations: the H_2_S group exhibited significantly higher pathological scores compared with the Control group, whereas XBJ pretreatment resulted in a substantial reduction in injury severity (Figure 2C). These findings are consistent with earlier studies demonstrating that toxic gas exposure can induce splenic structural damage and immune cell alterations.

Ultrastructural analysis by transmission electron microscopy (TEM) provided further insight into the nature of H_2_S-induced cellular injury. As shown in Figure 2D, splenocytes from the H_2_S group displayed characteristic features of pyroptosis, including mitochondrial swelling with vacuolization and disruption of cristae (red arrowheads). These mitochondrial abnormalities are consistent with the known capacity of sulfide to impair mitochondrial integrity and induce oxidative stress. In the H_2_S+XBJ group, such ultrastructural alterations were markedly reduced, with most mitochondria retaining relatively normal morphology, indicating that XBJ preserves mitochondrial structure and function during H_2_S exposure.

### 3.3 XBJ suppresses H_2_S-induced NLRP3 and caspase-1 expression in the spleen

Immunofluorescence staining was performed to evaluate the expression and distribution of NLRP3 and caspase-1 in splenic tissues. As depicted in Figure 3A, NLRP3 immunofluorescence (red) was barely detectable in the Control and XBJ groups, whereas H_2_S exposure elicited a robust increase in NLRP3-positive cells throughout the splenic parenchyma. Quantitative analysis of fluorescence intensity revealed that NLRP3 expression was significantly elevated in the H_2_S group compared with controls, while XBJ pretreatment markedly reduced this upregulation (Figure 3B).

**Figure 3:**
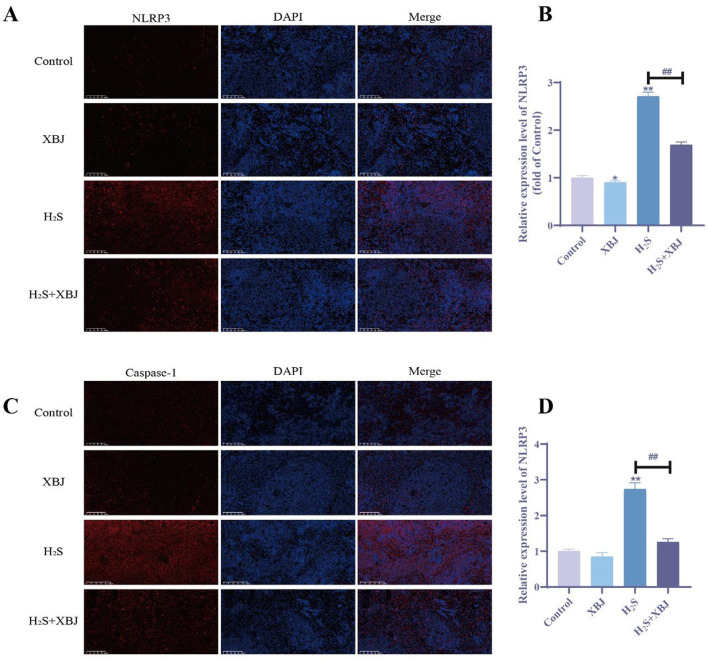
XBJ alleviates H_2_S-induced pyroptosis in rat spleen. **(A)** Representative immunofluorescence images showing NLRP3 expression (red) in splenic tissues. Nuclei were counterstained with DAPI (blue). Scale bar = 50 μm. **(B)** Quantitative analysis of NLRP3 fluorescence intensity normalized to the Control group. **(C)** Representative immunofluorescence staining for caspase-1 (red) in serial spleen sections. Scale bar = 50 μm. **(D)** Quantification of caspase-1 fluorescence intensity relative to Control. *p < 0.05 vs. Control group; **p < 0.01 vs. Control group; ^##^*p* < 0.01 vs. H_2_S group.

Similarly, caspase-1 immunofluorescence staining demonstrated negligible signal in Control and XBJ groups, but pronounced immunoreactivity following H_2_S exposure (Figure 3C). Quantification confirmed that caspase-1 fluorescence intensity was significantly higher in the H_2_S group, an effect that was substantially attenuated by XBJ pretreatment (Figure 3D). These findings indicate that H_2_S activates the NLRP3 inflammasome and promotes caspase-1 expression in the spleen, and that XBJ effectively suppresses this activation. The involvement of NLRP3 inflammasome in H_2_S-induced injury is consistent with recent reports demonstrating that sulfide exposure can trigger inflammatory cascades through NLRP3-dependent mechanisms.

### 3.4 XBJ modulates the NF-κB/NLRP3/caspase-1 signaling axis to counteract H_2_S-induced pyroptosis

To elucidate the molecular mechanisms underlying the protective effects of XBJ, we examined the expression of key proteins involved in the NF-κB and NLRP3/caspase-1 signaling pathways by Western blotting. As shown in Figure 4A, H_2_S exposure significantly upregulated the protein levels of NLRP3, ASC, and full-length GSDMD, accompanied by increased generation of cleaved GSDMD (GSDMD-N)—the pore-forming executor of pyroptosis. Additionally, processing of pro-caspase-1 to its active cleaved form (p20) was markedly enhanced in the H_2_S group. These changes were substantially reversed by XBJ pretreatment, indicating that XBJ inhibits H_2_S-induced inflammasome assembly and pyroptosis.

**Figure 4:**
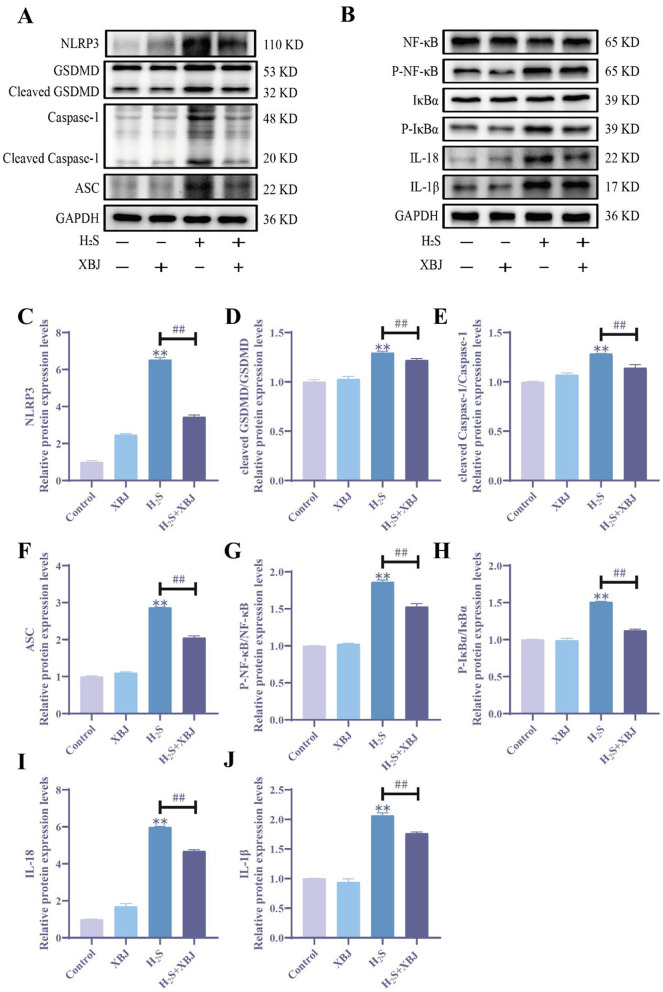
XBJ modulates NF-κB/NLRP3/Caspase-1 signaling axis to counteract H_2_S-induced splenic pyroptosis. **(A)** Representative immunoblots depicting protein expression levels of NLRP3 inflammasome components, including NLRP3, ASC, full-length and cleaved GSDMD (GSDMD-N), as well as pro- and cleaved caspase-1 (p20). **(B)** Representative Western blots showing phosphorylation status of NF-κB p65 (Ser536) and IκBα (Ser32), alongside total protein levels and downstream effectors IL-1β and IL-18. GAPDH served as the loading control. **(C–J)** Densitometric quantification of the indicated proteins relative to GAPDH or total protein (for phosphorylated forms). Data were normalized to control group values and are presented as mean ± SD from three independent experiments. **p < 0.01 vs. Control group; ^##^*p* < 0.01 vs. H_2_S group.

Assessment of the upstream NF-κB pathway revealed that H_2_S exposure induced phosphorylation of NF-κB p65 (Ser536) and IκBα (Ser32), indicating pathway activation (Figure 4B). Total protein levels of NF-κB p65 and IκBα remained relatively stable across groups. The downstream effectors IL-1β and IL-18 were also markedly elevated following H_2_S exposure, consistent with inflammasome activation and subsequent cytokine maturation. XBJ pretreatment significantly attenuated the phosphorylation of NF-κB p65 and IκBα and reduced the levels of IL-1β and IL-18 (Figure 4B).

Densitometric quantification of immunoblots (Figures 4C–J) confirmed that H_2_S exposure significantly increased the expression of NLRP3, ASC, GSDMD-N, cleaved caspase-1, and the ratios of phosphorylated to total NF-κB p65 and IκBα, as well as IL-1β and IL-18 levels. XBJ pretreatment effectively normalized these parameters, demonstrating its capacity to modulate the entire NF-κB/NLRP3/caspase-1 signaling axis. These findings align with accumulating evidence that XBJ exerts its anti-inflammatory effects through multitarget regulation of key signaling pathways, including NF-κB and NLRP3 inflammasome. The ability of XBJ to suppress both the priming (NF-κB-mediated) and activation (inflammasome assembly) phases of the pyroptotic cascade underscores its therapeutic potential in mitigating H_2_S-induced immunotoxicity.

## 4 Discussion

The present study demonstrates that acute inhalation exposure to 300 ppm H_2_S induces significant splenic injury in rats, characterized by elevated splenic coefficient, disrupted tissue architecture, ultrastructural features of pyroptosis, and oxidative stress imbalance. Mechanistically, H_2_S exposure activated the canonical NLRP3/caspase-1 signaling pathway, leading to GSDMD cleavage and subsequent release of pro-inflammatory cytokines IL-1β and IL-18, with concurrent activation of the upstream NF-κB pathway. Notably, pretreatment with XBJ injection (4 mg/kg for seven consecutive days) effectively attenuated H_2_S-induced splenic damage through inhibition of the NF-κB/NLRP3/caspase-1 axis, accompanied by restoration of antioxidant enzyme activities. These findings provide evidence that XBJ protects against H_2_S-induced immunotoxicity by suppressing pyroptotic cell death, highlighting its potential as a therapeutic intervention for H_2_S poisoning. To contextualize these observations, it is essential to first consider the established toxicological profile of H_2_S and the unique vulnerability of splenic tissue.

H_2_S has long been recognized as a mitochondrial toxin through its high-affinity inhibition of cytochrome c oxidase, disrupting electron transport chain and cellular respiration (42, 49, 50). However, accumulating evidence over the past decade indicates that H_2_S exposure also triggers robust inflammatory responses and immunotoxicity beyond its classical mitochondrial effects (19, 51, 52). The spleen, as the largest peripheral immune organ, is particularly vulnerable to circulating toxins and inflammatory mediators. In the present study, H_2_S exposure induced significant splenomegaly and elevated splenic coefficient, which were effectively reversed by XBJ pretreatment. These macroscopic changes correlated with histopathological alterations, including disrupted lymphoid follicles, white pulp atrophy, and extensive inflammatory cell infiltration—findings consistent with previous reports demonstrating that toxic gas exposure can induce splenic structural damage and immune cell alterations (33, 53). The observation that body weight remained unchanged across groups suggests that the observed splenic effects were direct consequences of H_2_S-induced local inflammation rather than secondary to systemic nutritional compromise.

The unique microarchitecture of the spleen merits specific consideration when interpreting these findings. Unlike other lymphoid organs, the spleen possesses an open circulatory system lacking a complete endothelial barrier, allowing direct exposure of resident lymphocytes to blood-borne toxins and inflammatory mediators (13, 54). This anatomical feature may render splenocytes particularly susceptible to H_2_S-induced pyroptosis, explaining the pronounced pathological changes observed in our study. Furthermore, the high density of immune cells within splenic compartments creates a microenvironment where paracrine signaling can amplify inflammatory responses, potentially converting initial toxin exposure into a self-propagating cycle of cell death and inflammation (55).

Oxidative stress serves as a critical trigger for NLRP3 inflammasome activation (21, 56, 57). In the present study, H_2_S exposure significantly depleted antioxidant reserves (GSH, SOD, CAT) while elevating lipid peroxidation marker MDA, indicating profound oxidative disturbance. These findings align with previous reports demonstrating that H_2_S-induced cytotoxicity involves ROS accumulation and antioxidant system compromise (42, 49, 58). The concentration-dependent duality of H_2_S biology is particularly relevant when interpreting these oxidative effects. At physiological concentrations, endogenous H_2_S functions as a cytoprotective gasotransmitter through protein persulfidation. Recent studies have demonstrated that low-concentration H_2_S inhibits pyroptosis via Rac1 thiolation (19) and attenuates oxidative stress-induced NLRP3 inflammasome activation through S-sulfhydration of c-Jun at Cys269 in macrophages, enhancing transcriptional activity on SIRT3 and p62 to reduce mitochondrial ROS production (51). However, the 300ppm exposure used in our study falls within the acutely toxic range, overwhelming cellular detoxification pathways and converting H_2_S from a protective mediator into a pro-oxidant, pro-pyroptotic agent. These seemingly contradictory findings underscore the critical influence of exposure concentration in dictating biological outcomes.

The NLRP3 inflammasome is a multiprotein complex that senses various danger signals and mediates caspase-1-dependent pyroptosis (59, 60). In the present study, H_2_S exposure significantly upregulated NLRP3 and ASC expression, promoted caspase-1 cleavage, and increased GSDMD-N generation—the pore-forming executor of pyroptosis. These findings are consistent with recent reports demonstrating that H_2_S exposure can trigger inflammatory cascades through NLRP3-dependent mechanisms (19, 61). The observed upregulation of IL-1β and IL-18 confirms functional inflammasome activation and downstream inflammatory consequences. Notably, the activation of NF-κB pathway—indicated by increased phosphorylation of p65 and IκBα–suggests its upstream regulatory role in priming the inflammasome by upregulating pro-IL-1β and NLRP3 expression. This aligns with the established two-signal model of NLRP3 inflammasome activation, where NF-κB-mediated transcriptional priming (signal 1) precedes inflammasome assembly and caspase-1 activation (signal 2) (57, 62, 63). The interplay between these pathways extends beyond simple linear activation, as recent evidence suggests that NLRP3 itself can modulate NF-κB activity through feedback mechanisms, creating a self-amplifying inflammatory loop that may drive the progressive tissue damage observed following H_2_S exposure.

XBJ injection, a traditional Chinese medicine preparation approved for sepsis treatment, has demonstrated therapeutic efficacy in various inflammatory conditions through multitarget regulation. Clinical evidence confirms that XBJ significantly reduces 28-day all-cause mortality in patients with sepsis (30), and preclinical studies have elucidated multiple mechanisms underlying its protective effects. Kang et al. (53) demonstrated that XBJ protects against sepsis-induced myocardial injury by inhibiting TLR4/NF-κB and JAK2/STAT3 signaling pathways, reducing inflammation and apoptosis. Chen et al. (33) reported that XBJ inhibits alveolar macrophage M1 polarization by regulating ROS-mediated NLRP3 inflammasome signaling. Zhang et al. (35) showed that XBJ alleviates LPS-induced acute lung injury through suppression of GSDME-mediated pyroptosis. Liu et al. (43) demonstrated that XBJ potently inhibits inflammasome activation in hepatocytes and ameliorates mouse liver ischemia-reperfusion injury, suggesting direct effects on inflammasome assembly beyond NF-κB modulation. Furthermore, a recent study revealed that XBJ exerts antioxidant effects by upregulating TRIM16 and NRF2 while inhibiting Keap1 expression, thereby activating the antioxidant response element pathway (64).

In our experimental system, XBJ pretreatment effectively suppressed H_2_S-induced NF-κB phosphorylation, diminished NLRP3 inflammasome assembly, and inhibited downstream pyroptosis. The capacity of XBJ to modulate both the priming (NF-κB-mediated) and activation (inflammasome assembly) phases of the pyroptotic cascade underscores its therapeutic promise. This dual mechanism holds particular relevance given that H_2_S toxicity encompasses both oxidative stress-driven NF-κB activation and direct inflammasome triggering. The restoration of antioxidant enzyme activities (GSH, SOD, CAT) by XBJ implies that its anti-pyroptotic effects may be partially mediated through preservation of redox homeostasis, consistent with its documented antioxidant properties (48, 64). The constituent compounds of XBJ—including hydroxysafflor yellow A, paeoniflorin, and ferulic acid—have individually demonstrated anti-inflammatory and antioxidant activities (65). Hydroxysafflor yellow A, derived from safflower, has been reported to attenuate ischemia-reperfusion injury by suppressing macrophage activation and mitigating oxidative stress (66). The ability of XBJ to simultaneously target oxidative stress, NF-κB activation, and inflammasome assembly positions it as a multi-pronged intervention capable of disrupting the self-amplifying inflammatory cycle triggered by H_2_S exposure. The protective effects of XBJ observed in our study likely involve interruption of the toxic cascade triggered by high-concentration H_2_S rather than enhancement of endogenous H_2_S signaling. This distinction carries important clinical implications, as therapeutic strategies for H_2_S poisoning must selectively target pathological pathways without interfering with beneficial H_2_S-mediated physiological functions. The concentration-dependent duality of H_2_S biology underscores this requirement, highlighting the need for interventions that discriminate between toxic and homeostatic signaling.

Several limitations of this study merit acknowledgment while also pointing toward future research directions. First, the sample size of four animals per group, while sufficient for basic parametric analyses (*n* ≥ 3) given the low intra-group variability observed and consistent with the 3R principle of Reduction in animal welfare, is relatively modest. Second, although we demonstrated that XBJ inhibits the NF-κB/NLRP3/caspase-1 pathway, the specific bioactive compounds responsible for this effect remain unidentified. XBJ contains multiple active constituents, and future studies employing network pharmacology approaches combined with compound knockout experiments could elucidate the precise molecular entities underlying its anti-pyroptotic effects. Third, our study focused on the canonical caspase-1-dependent pyroptotic pathway; however, emerging evidence implicates non-canonical pathways (caspase-4/5/11) and alternative inflammasomes (AIM2, NLRC4) in inflammatory cell death. Whether XBJ modulates these pathways warrants investigation. Fourth, although we observed reduced oxidative stress following XBJ treatment, distinguishing between direct antioxidant effects and indirect effects mediated through signaling pathway modulation requires further clarification using targeted approaches. Fifth, our study utilized a single time point (12 h post-exposure); time-course experiments would better characterize the temporal dynamics of pyroptotic signaling and establish optimal intervention windows for XBJ administration. Sixth, while the 300 ppm H_2_S exposure model recapitulates acute poisoning scenarios, chronic low-dose exposure models would provide complementary insights into occupational and environmental H_2_S toxicity, where cumulative low-level exposure may produce distinct pathological outcomes.

In summary, our findings demonstrate that XBJ injection confers protection against H_2_S-induced splenic injury by suppressing the canonical caspase-1/NLRP3 pyroptotic pathway through inhibition of upstream NF-κB signaling and restoration of antioxidant equilibrium. These results provide novel insights into the immunotoxic mechanisms of H_2_S poisoning and identify XBJ as a potential therapeutic strategy for mitigating H_2_S-induced immune organ damage. The capacity of XBJ to modulate multiple nodes of the pyroptotic cascade—oxidative stress, NF-κB activation, inflammasome assembly, and caspase-1 cleavage—underscores its therapeutic potential as a multi-target intervention for H_2_S toxicity. Given XBJ's established clinical safety profile and approval for sepsis management, repurposing this agent for H_2_S poisoning represents an attractive translational opportunity. Future research should focus on identifying specific bioactive components responsible for these protective effects, establishing optimal intervention windows through time-course studies, and evaluating efficacy in chronic exposure models relevant to occupational and environmental settings.

## 5 Conclusion

In conclusion, this study provides the first evidence that XBJ injection protects against H_2_S-induced splenic pyroptosis in rats by suppressing the canonical caspase-1/NLRP3 inflammasome pathway via inhibition of upstream NF-κB signaling (Figure 5). These findings offer new insights into the immunotoxic mechanisms of H_2_S poisoning and highlight XBJ as a promising therapeutic candidate for H_2_S-induced immune organ damage.

**Figure 5:**
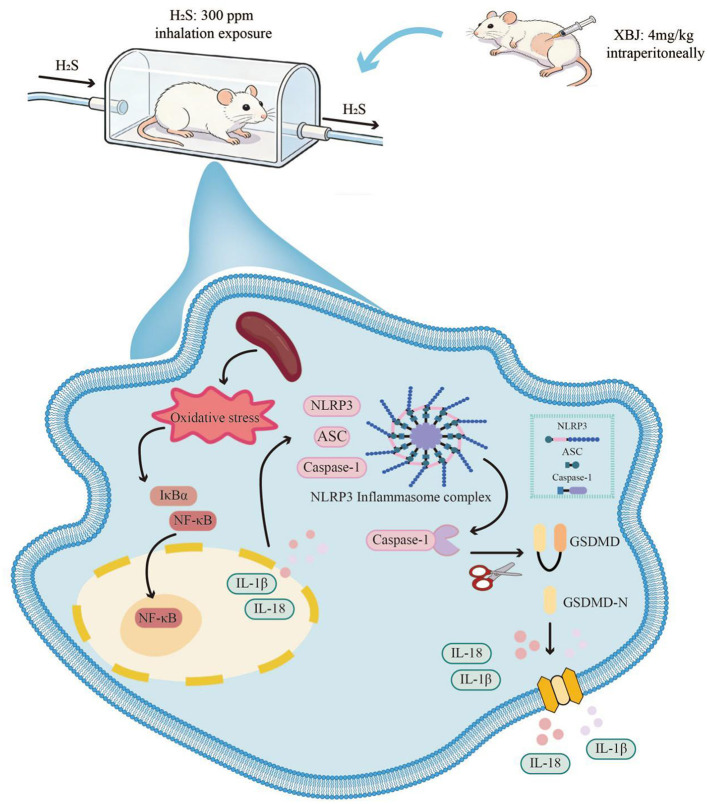
Schematic illustration of the proposed mechanism by which XBJ alleviates H_2_S-induced splenic pyroptosis in rats.

## Data Availability

The original contributions presented in the study are included in the article/Supplementary material, further inquiries can be directed to the corresponding authors.
