# Supplementary material for: Xuebijing alleviates acute hydrogen sulfide-induced pyroptosis in rat spleen via suppression of the caspase-1/NLRP3 pathway
Source: Front Vet Sci. 2026 Jul 29;13:1914569. doi: 10.3389/fvets.2026.1914569 (PMC13461396; doi:10.3389/fvets.2026.1914569)
Supplement: Supplementary file 1 [file Data_Sheet_1.pdf]

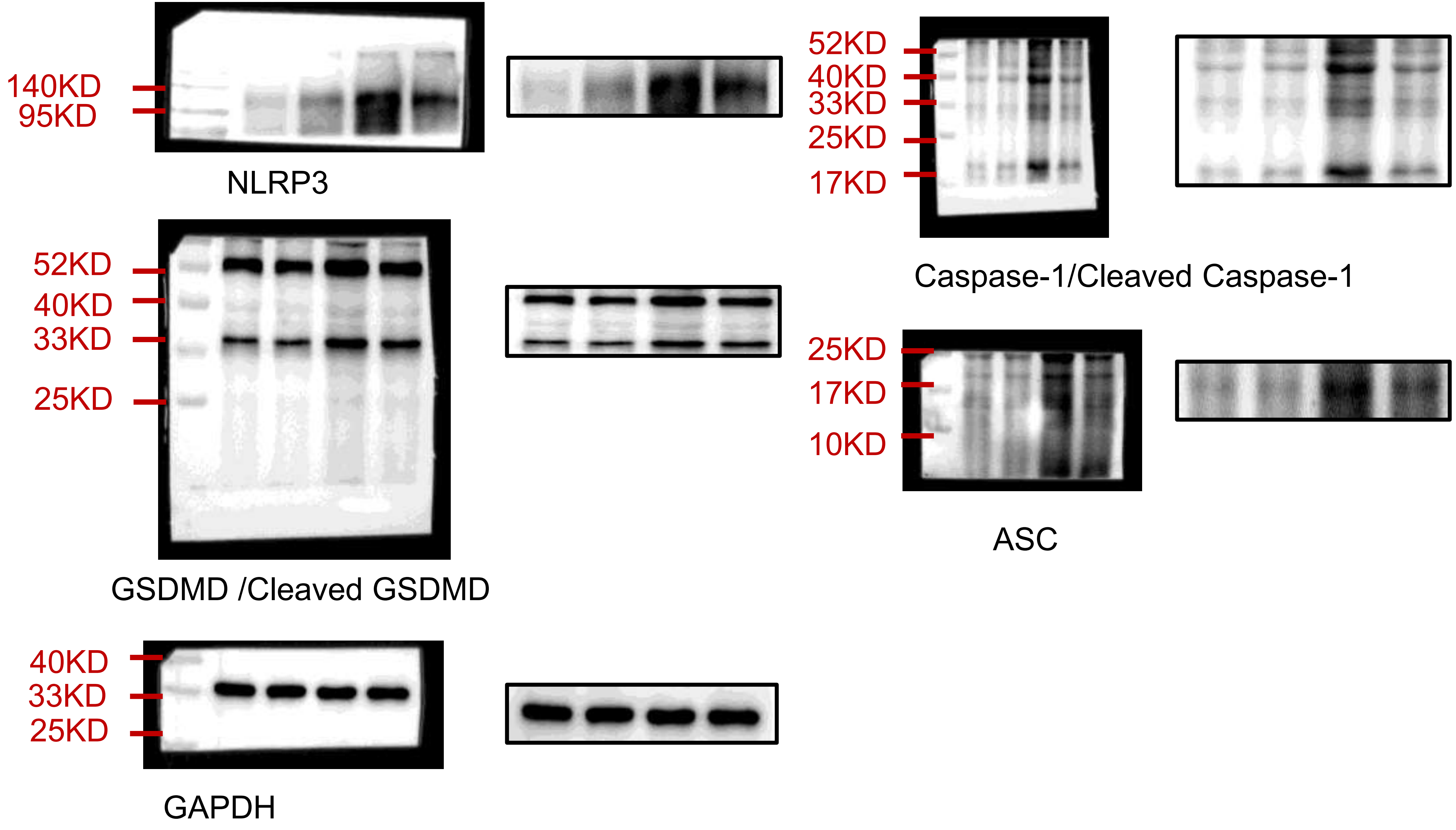
140KD
95KD
NLRP3
52KD
40KD
33KD
25KD
17KD
Caspase-1/Cleaved Caspase-1
52KD
40KD
33KD
25KD
GSDMD /Cleaved GSDMD
25KD
17KD
10KD
ASC
40KD
33KD
25KD
GAPDH

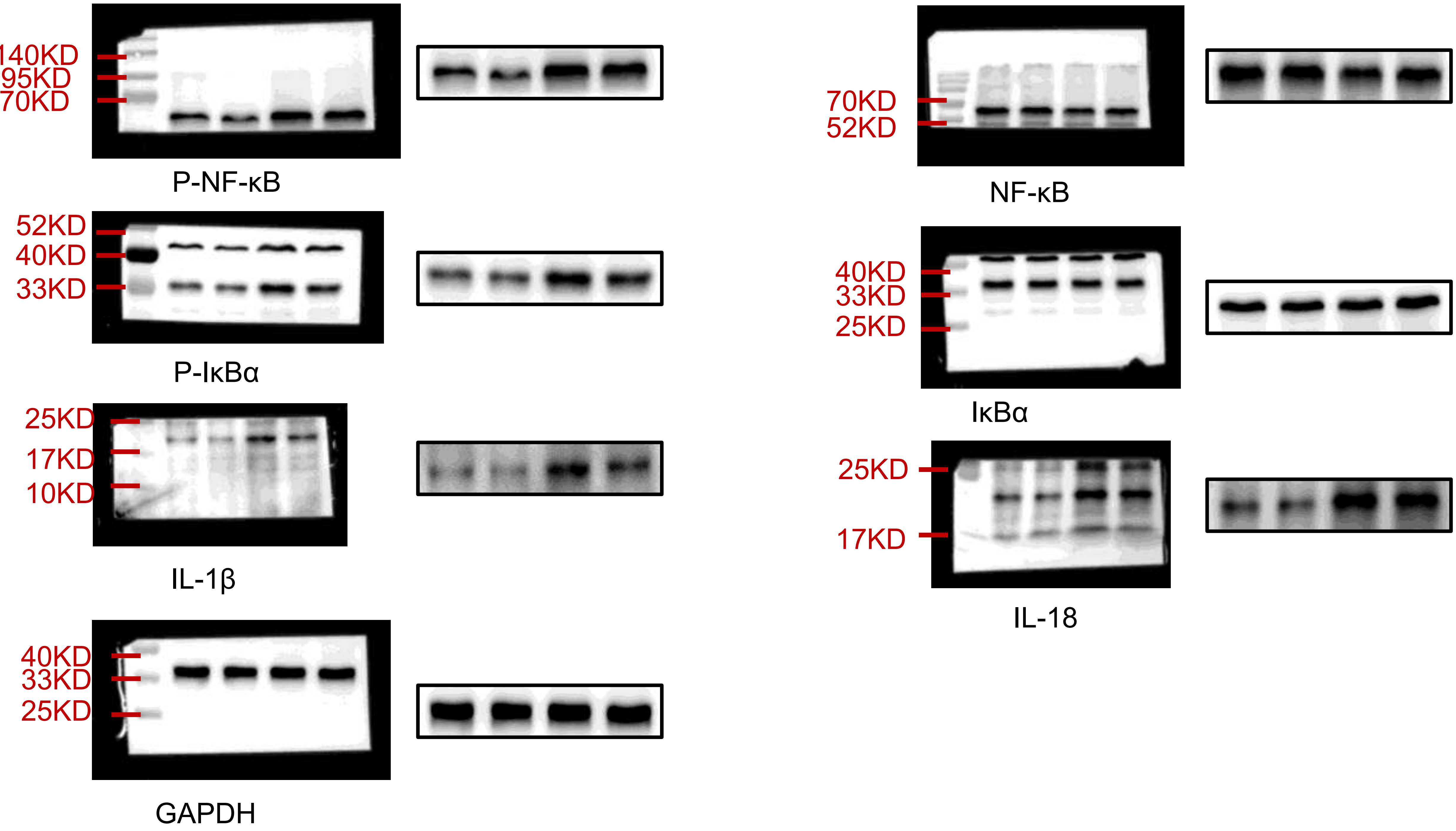
140KD
95KD
70KD
P-NF-κB
70KD
52KD
NF-κB
52KD
40KD
33KD
P-IκBα
40KD
33KD
25KD
IκBα
25KD
17KD
10KD
IL-1β
25KD
17KD
IL-18
40KD
33KD
25KD
GAPDH

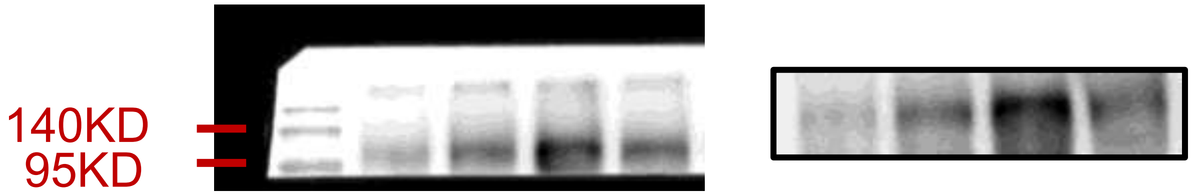

NLRP3 2

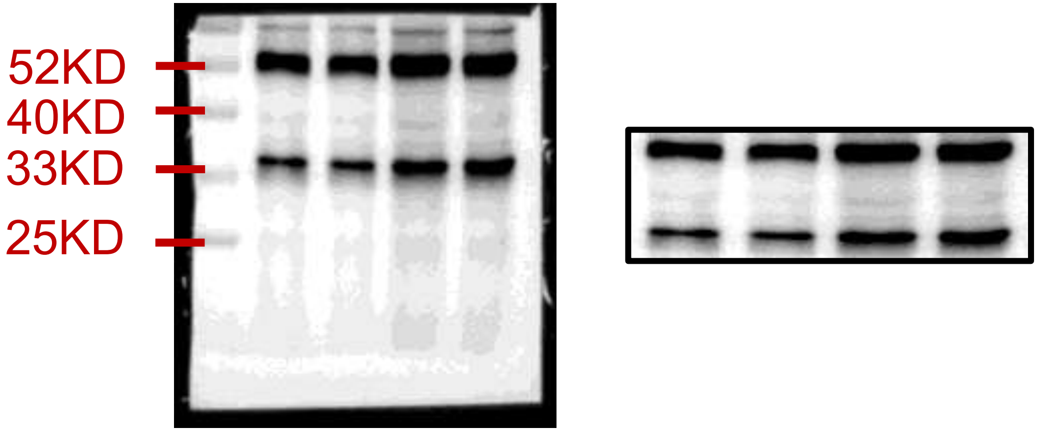

GSDMD /Cleaved GSDMD 2

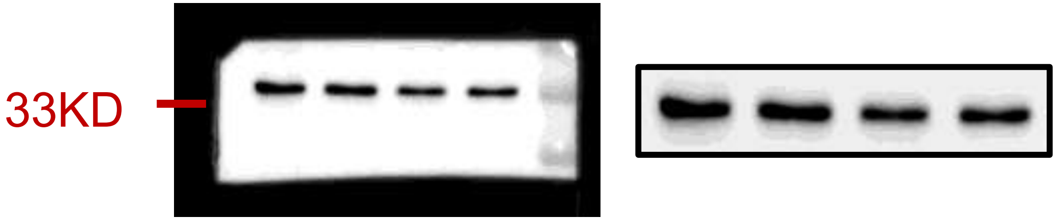

GAPDH 2

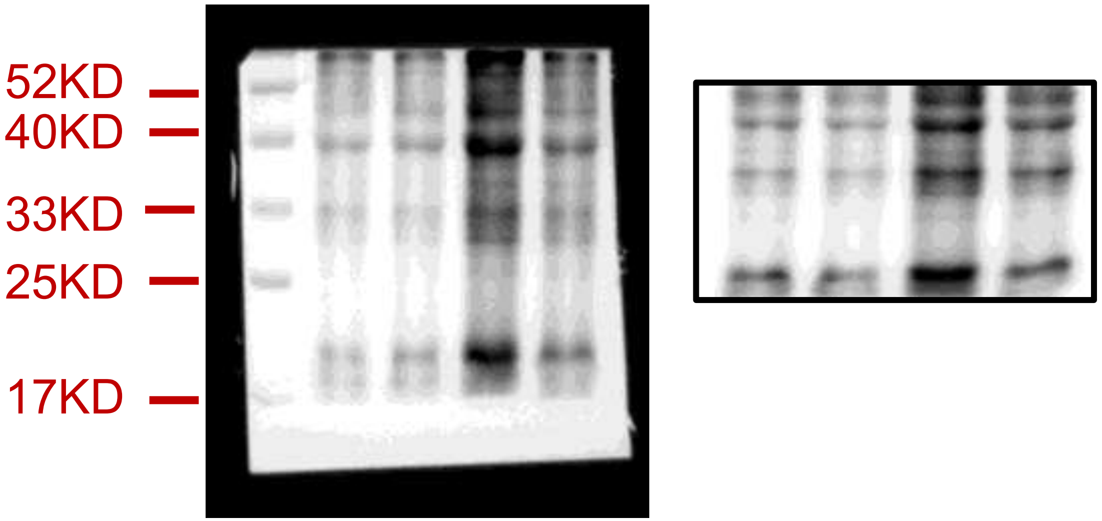

Caspase-1/Cleaved Caspase-1 2

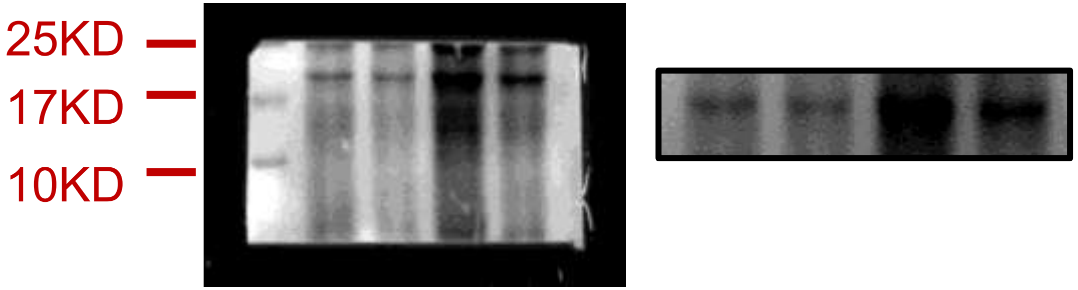

ASC 2

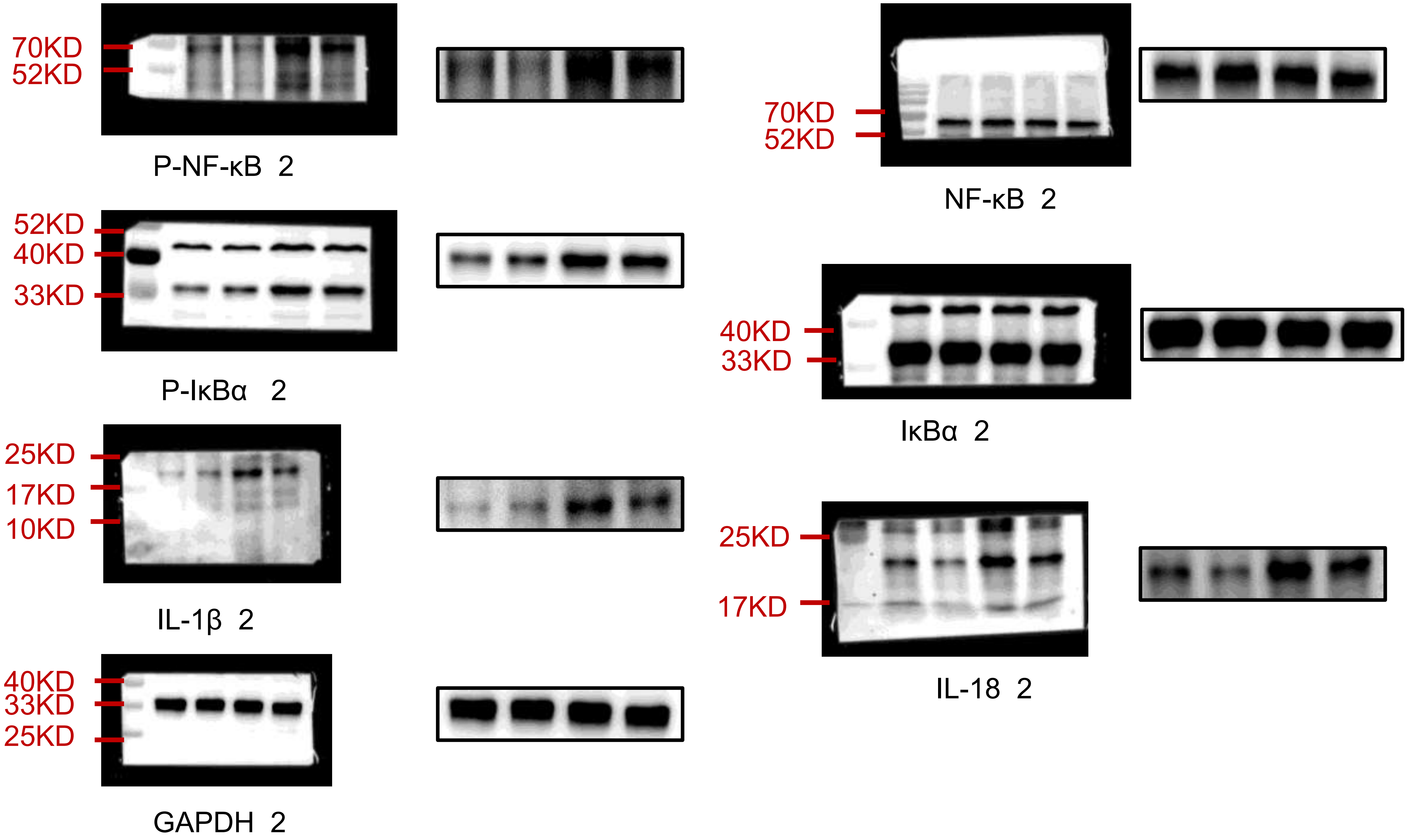
70KD
52KD
P-NF-κB 2
70KD
52KD
NF-κB 2
52KD
40KD
33KD
P-IκBα 2
40KD
33KD
IκBα 2
25KD
17KD
10KD
IL-1β 2
25KD
17KD
IL-18 2
40KD
33KD
25KD
GAPDH 2

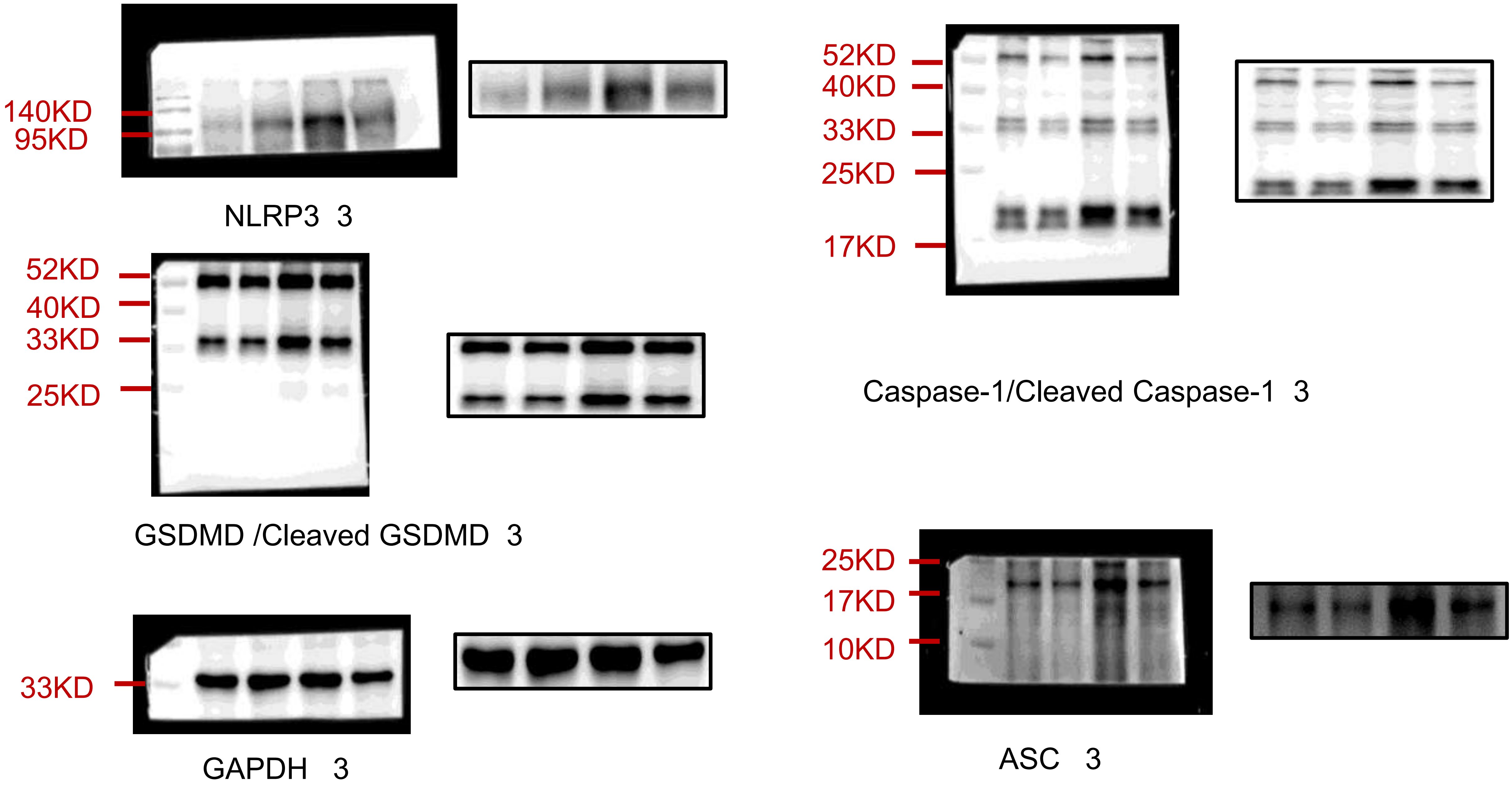
140KD
95KD
NLRP3 3
52KD
40KD
33KD
25KD
GSDMD /Cleaved GSDMD 3
33KD
GAPDH 3
52KD
40KD
33KD
25KD
17KD
Caspase-1/Cleaved Caspase-1 3
25KD
17KD
10KD
ASC 3

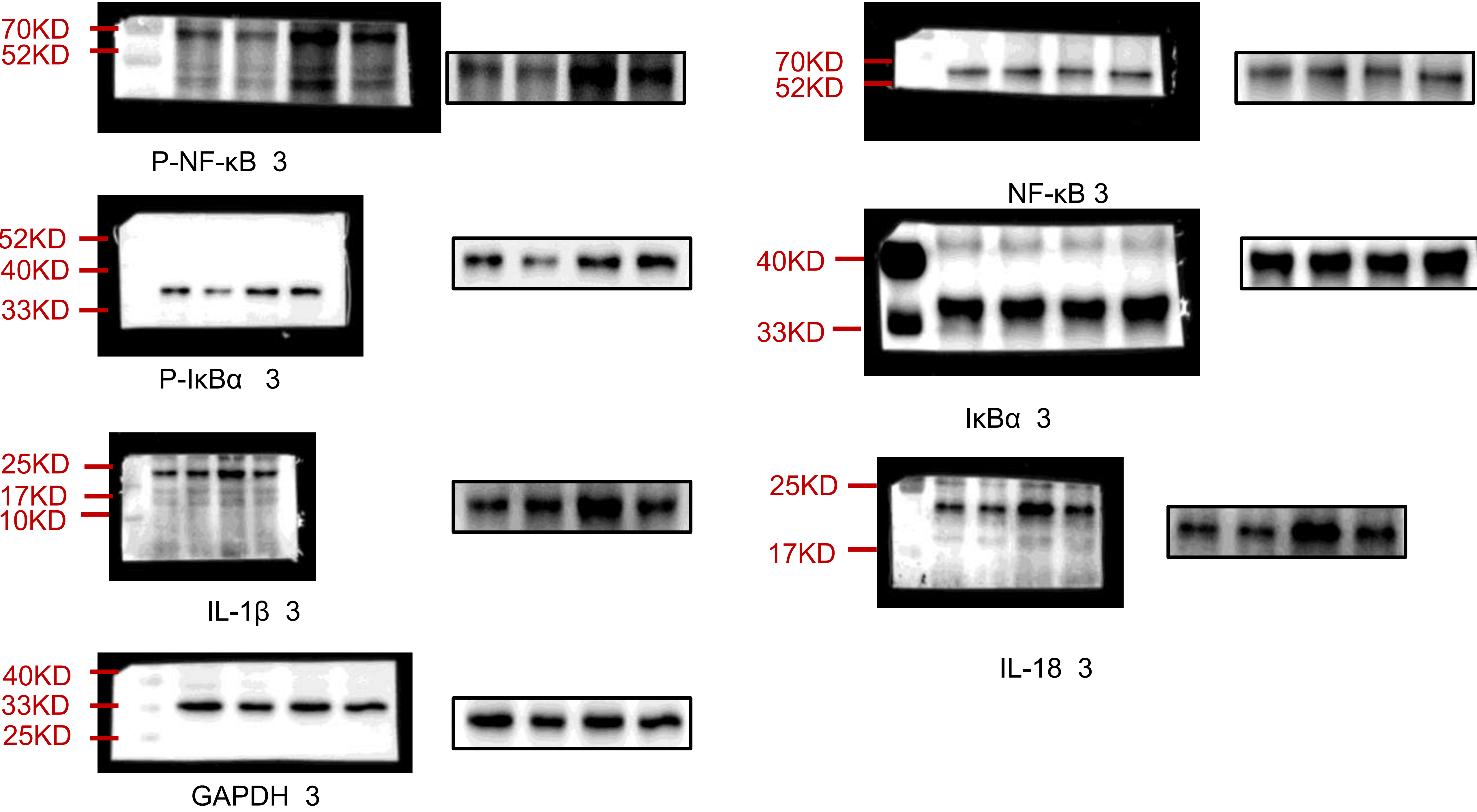
70KD
52KD
P-NF-κB 3
70KD
52KD
NF-κB 3
52KD
40KD
33KD
P-IκBα 3
40KD
33KD
IκBα 3
25KD
17KD
10KD
IL-1β 3
25KD
17KD
IL-18 3
40KD
33KD
25KD
GAPDH 3
